# Prehabilitation in Adult Solid Organ Transplant Candidates

**DOI:** 10.1007/s40472-023-00395-4

**Published:** 2023-03-25

**Authors:** Evelien E. Quint, Manoela Ferreira, Barbara C. van Munster, Gertrude Nieuwenhuijs-Moeke, Charlotte te Velde-Keyzer, Stephan J. L. Bakker, Coby Annema, Sunita Mathur, Robert A. Pol

**Affiliations:** 1 Division of Transplantation Surgery, Department of Surgery, University Medical Center Groningen, University of Groningen, P.O. Box 30 001, 9700 RB Groningen, The Netherlands; 2 Department of Physical Therapy, University of Toronto, Toronto, ON Canada; 3 Division of Geriatric Medicine, Department of Internal Medicine, University Medical Center Groningen, University of Groningen, Groningen, the Netherlands; 4 Department of Anesthesiology, University of Groningen, University Medical Center Groningen, Groningen, the Netherlands; 5 Division of Nephrology, Department of Internal Medicine, University Medical Center Groningen, University of Groningen, Groningen, the Netherlands; 6 Division of Nursing Science, Department of Health Sciences, University Medical Center Groningen, University of Groningen, Groningen, The Netherlands; 7 School of Rehabilitation Therapy, Queen’s University, Kingston Ontario, Canada

**Keywords:** Prehabilitation, Transplant, Outcomes

## Abstract

**Purpose of Review:**

To highlight the importance of biological age in the context of prehabilitation and to present relevant research regarding prehabilitation prior to solid organ transplantation.

**Recent Findings:**

Studies on the effect of prehabilitation have been performed in kidney-, lung-, liver-, and heart transplant patient populations. Prior to kidney transplantation, exercise interventions have been shown to improve cardiopulmonary- and physical fitness and result in a decreased length of hospital stay postoperatively. Among lung transplant candidates, various methods of prehabilitation have been studied including home-based, outpatient and in-patient programs, consisting of physical training, psychological support, education, and nutritional interventions. Overall, prehabilitation seems to improve or maintain quality of life and exercise capacity in this patient population. Patients undergoing liver transplantation seem to benefit from prehabilitation as well. Not only does it seem safe and feasible, but significant improvements in aerobic and functional capacity have also been found. Regarding heart transplant candidates, both inpatient and outpatient, supervised prehabilitation programs show promising results with improvements in exercise capacities and quality of life.

**Summary:**

Prehabilitation is an effective and safe intervention for improving functional outcomes of solid organ transplant patients. Future studies should evaluate whether prehabilitation translates into improved pre- and post-transplant clinical outcomes.

## Introduction

Solid organ transplantation (SOT) is the preferred treatment option for patients diagnosed with end stage organ failure [1]. Every year, more than 100,000 SOTs are performed worldwide [2]. Although in many cases lifesaving, a substantial number of patients experience postoperative adverse events or complications which may impact short- and long-term graft and patient outcomes. A range of factors are associated with increased risk of pre-, peri-, and post-operative complications including older age, comorbidities and frailty [3]. Furthermore, lifestyle and behavioral aspects such as physical inactivity, malnutrition, and psychological issues may increase this risk.

Major surgeries have shown to reduce physiological and functional capacity by up to 40%, partly due to pre-operative inactivity, leading to a reduced reserve capacity and postoperative immobility leading to muscle deterioration and atrophy [4]. These factors give rise to a decreased ability to cope with stressors such as a SOT and to obtain allostasis. In this perspective it is necessary for SOT candidates to be in an optimal state of health prior to transplantation to increase resilience and mitigate post-operative complications [5, 6].

The physiological stress of a major surgical procedure can be compared to running a marathon which requires training to complete the course and prevent complications. Prehabilitation aims to increase fitness and wellbeing and increase the physiological reserve capacity in the preoperative period through the implementation of exercise, dietary interventions, cognitive interventions, and psychosocial interventions to prevent declines across multiple reserves [7]. By intervening prior to surgery, behavioral and lifestyle risk factors can be modified in order to improve patients’ physiologic reserve and prepare them for the surgery and post-surgical recovery.

To date, older adults (> = 65 years) are the most rapidly growing age group undergoing kidney-, liver-, heart-, and lung transplantation [8–11]. For example, among kidney transplant patients, the number of older adults receiving a kidney has tripled in the last decade, with older adults now comprising almost one-fifth of the kidney transplant population [12, 13]. Similarly, the proportion of older adults in the lung transplant recipient population has increased from 3% to approximately 35% in the past 2 decades [11].

Predictably, surgery in older patients is associated with additional risks [14]. Older age at the time of transplantation is associated with adverse outcomes across all SOT populations such as an impaired long-term survival and a higher risk of developing complications such as infections postoperatively [15–19]. The impact of age, however, depends on distinguishing healthy, chronological aging from biological aging. Aging-associated syndromes, such as sarcopenia, impaired cognition, multi-morbidity, and poor nutrition, have been shown to occur in all chronologic age groups. Even young patients with end-stage organ disease can develop frailty, as a result of which frailty could better be described as associated with biological, as opposed to chronologic age.

Previous studies show that prehabilitation is feasible, effective and safe in patients undergoing major abdominal surgery [20, 21]. The evidence regarding the effect, feasibility, and safety of prehabilitation in the SOT population remains limited, most likely to the enormous heterogeneity in populations, interventions and targeted domains. Interventions vary in intensity, duration, and exercise modality due to the differing functional status of patients. Some programs consist of physical training only, while others additionally offer multi-modal interventions including nutritional support and psychological support.

In SOT, a window of opportunity to intervene exists preoperatively due to the waitlist period. Though waitlist time is variable, ample time is available for an intervention. This time could be used to implement lifestyle interventions and improve clinical outcomes. In this review, we will highlight the most relevant, up-to-date literature regarding the physiological impacts of prehabilitation in adult SOT candidates and elucidate knowledge gaps to guide future research.

## The Impact of Biological Aging on Transplant Outcomes

The concept of biological aging is of great importance in the context of prehabilitation. The term biological aging encompasses frailty, sarcopenia, and metabolic alternations. It is the progressive decline in physiological reserve capacity due to the accumulation of cellular damage that occurs as a person gradually accumulates cell damage over time. It differs from chronological aging and takes multiple factors into account, such as metabolic-, nutritional-, and physical changes [22]. Biological aging can clinically manifest as frailty and one way to measure biological age is by using a frailty measurement as a proxy [23]. Frailty is a multidimensional, age-associated condition associated with declines in physical, psychological, cognitive, and immune reserves [24]. These deficits result in a patient’s diminished ability to cope with acute and chronic stressors and to obtain/maintain homeo- or allostasis under a variety of conditions.

Although chronological age cannot be slowed or reversed, biological aging can be slowed down through lifestyle interventions. This may be the key to improving the health and wellbeing of especially older and/or frail patients within the SOT population.

Frailty is prevalent among SOT recipients. One in six kidney transplant patients is considered frail. This is associated with adverse outcomes such as delayed graft function, poorer tolerance to immunosuppression, (prolonged duration of) hospitalization, graft loss, and mortality [12, 25–30]. The prevalence of frailty in lung transplant patients ranges from 10 to 70% and leads to a higher risk of mortality, prolonged hospitalization, rehospitalization, and a decrease in health-related quality of life [17, 31–35]. In the liver transplant population, 25% of transplant patients are considered frail which is associated with an increased mortality rate, number of hospitalizations and length of stay in the hospital [36–39]. One-third of heart transplant patients experience frailty prior to transplantation which is associated with all-cause mortality and a trend toward longer ICU and hospital stays [40]. To improve the physiological reserve capacity/frailty status and therefore clinical outcomes, an intervention catered to those able to reverse their condition (pre-frail) must be implemented in transplant care.

One of the constituents of frailty is sarcopenia. Sarcopenia is defined as a syndrome characterized by a progressive and generalized loss of skeletal muscle mass and strength. It is associated with increased risk of adverse outcomes [41]. Skeletal muscle is one of the main tissues that counters the injury and stress response induced by surgery [42]. Therefore, a patient must have a certain amount of muscle mass to have the reserve capacity to fight physiological stressors, and to increase the chances of better post-operative outcomes. Among kidney transplant patients, metabolic alterations, dialysis, and the associated sedentary lifestyle contribute to the development of sarcopenia [43]. Consequently, the incidence of sarcopenia is far greater in patients with end-stage renal disease (ESRD) and kidney transplantation candidates than in the general population (15–21%) [44]. In lung transplant candidates, sarcopenia is associated with increased disability, prolonged ventilation, and prolonged hospital stay after transplantation [45, 46]. The same holds true for liver transplant candidates, where sarcopenia is associated with adverse outcomes such as mortality [47]. In the heart transplant population, sarcopenia is associated with post-transplant infections within 6 months following transplantation [48].

## Prehabilitation

Prehabilitation can be implemented prior to surgery to improve pre- and post-operative outcomes. It is a term used to describe an intervention aimed at optimizing a patient’s overall fitness before an operation. This is especially relevant in the SOT population as almost half of SOT patients live a sedentary lifestyle [48]. The following sections will outline evidence for prehabilitation prior to kidney-, lung-, liver-, and heart transplantation. See Table 1 for summary of interventions in each organ group.

**Table 1:** Interventions and outcomes measured in prehabilitation for kidney transplant candidates

Author, year	Intervention	Location	Supervision	Frequency	Duration (weeks)	Outcomes
KIDNEY						
McAdams-DeMarco 2019 [26]	Physical training -Breathing exercises -Stretching -Aerobic exercise -Strength training	Outpatient clinic	Physical therapist	Weekly (1h)	8	-% Change in mean activity counts per minute -Post-transplant LOS -(Severe) adverse events -Participation -Satisfaction
Lorenz 2020 [50]	Physical training -Endurance training -Strength training -Flexibility training	Outpatient clinic	Physical therapist	Twice weekly (1h)	8	-FFP -SPPB -KDQOL-SF -Alternate measures of muscle mass -(Severe) adverse events -Vital signs -Acceptability -Participation
Ma 2022 [51]	Physical training -Aerobic exercise -Functional resistance training -Post-exercise session stretching	Home-based	Remote guidance by experimenter	Variable	12	-6MWT -5R-STS -SF-36 -Grip strength -4-m gait speed -HADS
LUNG						
Li 2013 [53]	Physical training -Stretching -Aerobic exercise (arm ergometer, cycle ergometer, treadmill training) -Resistance training (strengthening exercises)	Outpatient clinic	Physical therapist and physical therapy assistant	Thrice weekly (1, 5–2 h)	Variable (entire waitlist period)	-6MWD -6MWT -SF-36 -SGRQ -EQ-5D -Visual analog scale -Standard Gamble -Discharge disposition -LOS in the ICU and hospital -Length of time intubated
Florian 2013 [54]	Physical training -Breathing exercises -Strengthening exercises -Aerobic training Nutritional counseling Social assistance Educational lectures	Outpatient clinic	Physical therapist	Thrice weekly (1, 5 h)	12	-6MWT -SF-36 -SpO_2_ -Modified Borg Scale
Wickerson 2020 [55]	Physical training -Stretching -Functional exercises -Resistance training -Aerobic exercise	Outpatient clinic	Physiotherapist	Thrice weekly (1, 5 h)	Variable (entire waitlist period)	-6MWT -SPPB -5R-STS -Balance score -4-m walk score -Gait speed -Tandem time -Chair stands score
Pehlivan, 2018 [56]	Physical training -Aerobic training -Strengthening exercises Patient education	2 days of group exercises at out-patient clinic/3 days of home-based exercise	Physiotherapist/ no-supervision	5 times weekly	At least 8	-6MWT -FVC -FEV_1_ -mMRC -Muscle strength -BDI -SF-36
Pehlivan, 2018 [57]	Physical training -Aerobic training -Strengthening/resistance training -Breathing exercises	2 days of group exercises at out-patient clinic/3 days of home-based exercise	Physiotherapist/ no-supervision	5 times weekly		-Maximum inspiratory pressure—maximum expiratory pressure -6MWT -mMRC -Pulmonary function test -Carbon monoxide diffusion test
Jastrzebski 2006 [58]	Physical training -Correctional exercise -Movements of the thorax -Isometric exercise -Respiratory muscle exercise -Bicycle ergometer training -Running	Inpatient (4 weeks)/Out-patient thereafter	Instructor of rehabilitation/ no-supervision	Daily (30 min)	6	-MRC -OCD -BDI -Borg Scale SF-36 -SGRQ
Gloeckl 2012 [59]	Physical training -Endurance training (Interval training OR continuous training) Breathing therapy Education Psychological support	Inpatient	Supervised	5–6 times weekly (10–36 min)	3	-6MWD -Modified Borg scale -PWR -Pulmonary function -SF-36 -Feasibility
Kenn 2015 [60]	Pulmonary rehabilitation Education	Inpatient	Supervised	5–6 times weekly (10–45 min)	5	-6MWD -SF-36 -Pulmonary function test -Blood gases
Massierer 2020 [61]	Physical training -Warm-up -Aerobic training -Strengthening exercises -Stretching exercises Cool down exercises	Home-based	Unsupervised	Up to 5 times weekly (up to 30 min)	Variable (entire waitlist period)	-6MWD
LIVER						
Limongi 2014 [63]	Physical training -Cough and breathing exercises -Isometric force exercises	Home-based	Unsupervised	Daily	12	-BMI -MIP -MEP -EMG -CPET -SF-36
Wallen 2019 [64]	Physical training -Aerobic training (cycle ergometer or walking) -Resistance strength exercise (circuit-based with weights)	Home-based /Hospital gym	Supervised/ unsupervised	Thrice weekly	8	-(Severe) adverse events -Attendance -CPET -6MWD -Grip strength dynamometer -Global muscular strength -CLDQ
Chen 2020 [65]	Physical training -Walking training by increase daily step-goal Nutritional support	Home-based	Unsupervised, weekly supervised counseling and daily motivational phone calls	5 times weekly (30 min)	12	-CPET -Anthropometrics -Bioelectrical impedance -DXA -CT body composition -SIP
Debette-Gratien 2015 [66]	Physical training -Aerobic training (cycle ergometer) -Muscle strength exercise (press body building type)	In-hospital gym	Supervised	Twice weekly	12	-Aerobic capacity -Maximal power -Muscle strength -Threshold power -Cardiac frequency -SF-36 -6MWT -Acceptability -(Serious) adverse events
Williams 2019 [67]	Physical training -Functional resistance exercises (video guide) -Aerobic exercises (video guide) -Walking program (daily step targets)	Home-based	Unsupervised, weekly supervision via telephone	Twice weekly (20 min)	12	-(Serious) adverse events -Adherence -ISWT -SPPB -ADS count -HADS -EQ-5D-5L -EQ-VAS
Morkane 2020 [68]	Physical training -Aerobic training (cycle ergometer)	Hospital gym	Supervised	Thrice weekly (40 min)	6	-CPET -Anthropometrics -Handgrip strength -RFH Global Assessment Data Collection Form -Donor risk index -LOS ICU -Hospital LOS -Survival (6 months)
Al-Judaibi 2019 [69]	Physical training -Aerobic training (cycle ergometer) -Resistance strength exercise Education regarding physical activity Nutritional support	Hospital gym/ At home	Supervised/unsupervised with twice/thrice weekly phone calls	1–5 times weekly	Variable (until suitable for transplantation)	-90-day readmission post- transplant -Hospital LOS post-transplant -Postoperative complications -Mortality
Lin 2021 [70]	Physical training -Force: weights/resistance bands -Aerobic: treadmill, elliptical or stationary bikes	Home-based Rarely: outpatient physical therapy	Unsupervised, follow-up appointment once a month Rarely: supervised	5 times weekly (30 min)	Variable (until transplantation)	-LFI -6MWT -GST -Compliance
HEART						
Gimeno-Santos 2020 [71]	Physical training -Interval endurance training (stationary bicycle) -Strength training -Breathing exercises Nutritional intervention -Personalized dietary plan -Whey protein supplementation Mindfulness-based stress reduction	Outpatient gym facility	Supervised	Twice weekly (1 h)	8	-6MWD -STS -YPAS -Exercise capacity -MLHFQ -HADS
Taya 2018 [72]	Physical training -Aerobic exercise training (bicycle ergometer) -Resistance training	In-patient clinic	Supervised	3–4 times per week (5–20 min)	Variable	-Handgrip strength -Knee extensor strength -(Serious) adverse events
Forestieri et al. [73]	Physical training -Breathing exercises and global active exercises OR -Intermittent training	In-patient clinic	Supervised	Twice daily	Variable	-6MWT -MIP

*LOS*, length of stay; *FFP*, Fried Frailty phenotype; *SPPB*, short physical performance battery; *KDQOL-SF*, kidney disease quality of lifeshort form; *6MWT*, 6-min walk test; *5R-STS*, 5 repetition-Sit-to-stand test; *SF-36*, short form health survey; *HADS*, hospital anxiety and depression scale; *6MWD*, 6-min walk distance; *SGRQ*, St. George’s Respiratory Questionnaire; *EQ-5D*, EuroQol-5D; *ICU*, intensive care unit; *FVC*, forced vital capacity; *FEV*_*1*_, forced expiratory volume in one second; *mMRC*, modified medical research council dyspnea scale; *BDI*, beck depression inventory; *MRC*, medical research council scale; *OCD*, oxygen cost diagram; *BDI*, baseline dyspnea index; *PWR*, peak work rate; *BMI*, body mass index; *MIP*, maximal inspiratory pressure; *MEP*, maximal expiratory pressure; *EMG*, electromyography; *CPET*, cardiopulmonary exercise testing; *CLDQ*, chronic liver disease questionnaire; *DXA*, dual-energy X-ray absorptiometry, *CT*, computed tomography; *SIP*, sickness impact profile; *ISWT*, incremental shuttle walk test; *ADS*, average daily step; *EQ-5D-5L*, EuroQol 5-dimension 5-level; *EQVAS*, EuroQoL visual analogue scale; *RFH*, royal free hospital; *LFI*, liver frailty index; *GST*, gait speed test; *STS*, sit to stand; *YPAS*, Yale Physical Activity Survey; *MLHFQ*, Minnesota Living with Heart Failure Questionnaire

### Prehabilitation and Kidney Transplantation

Three intervention studies focusing on the effect of prehabilitation prior to kidney transplantation have been performed. In a study by Mcadams-Demarco and colleagues [49•], 18 kidney transplant candidates were enrolled in a single-arm intervention trial of which one-third of participants were considered frail. The prehabilitation program consisted of weekly, 1-h, physical activity training with a physical therapist. After 2 months of prehabilitation, a 64% improvement in physical activity measured using an accelerometer was seen. Four participants underwent transplantation during the study and exhibited a significantly decreased length of hospital stay after transplantation compared to historical controls. Furthermore, no adverse events were reported [49•]. A study by Lorenz et al. examined the effects of an 8-week exercise intervention prior to kidney transplantation. A total of 19 participants took part in the exercise intervention consisting of endurance, strength, and flexibility training. The supervised exercise sessions took place twice a week, for 1 h. Participants who received dialysis were scheduled on non-dialysis days. After 8 weeks of exercise intervention, improvements were seen in frailty status measured by the Fried Frailty phenotype (38.1% vs. 23.8% of participants were frail, *p* = 0.18), though not statistically significant. However, they concluded that exercise based prehabilitation seems acceptable, safe, and feasible and is associated with significant changes in fatigue, grip strength, physical activity, walking speed, and balance [50]. Recently, Ma et al. published the results of their randomized clinical trial (RCT) on remote, supervised, home exercise prehabilitation to improve physical function in (*n* = 21 in intervention arm). After 12 weeks of personalized intervention, consisting of aerobic exercise, functional resistance training, and post-exercise session stretching, they found significant improvements in cardiopulmonary and physical fitness as shown by the 6-min walk test, 5-times-sit-to-stand test, and 4-m walking speed. Unlike the other two studies, these patients performed prehabilitation exercises at home, under remote supervision. Interestingly, acceptance was high among participants, based on 94% of participants who met the eligibility criteria agreed to participate in this trial and 75% completed the entire intervention. Therefore, at-home prehabilitation exercise for kidney transplant candidates seems acceptable, with good adherence [51].

Regarding ongoing prehabilitation studies in the kidney transplant population, only one study protocol has been published thus far. Perez-Saez et al. started their RCT in 2020, which investigates the effect of multi-modal prehabilitation on adverse outcomes 90 days post kidney transplant in 38 frail and 76 non-frail kidney transplant candidates. The intervention group will receive individually tailored, multimodal prehabilitation including physical exercise, nutritional supplementation, and psychological advice [52]. The study will test the effect of this prehabilitation program on the primary outcome which is a composite measure of adverse outcomes at day 90 post-kidney transplantation including delayed graft function, readmission, surgical complications, and all-cause death.

### Prehabilitation and Lung Transplantation

Several studies have explored the effects of prehabilitation in lung transplant candidates. One large retrospective cohort analysis by Li et al. showed that pre-operative pulmonary rehabilitation consisting of stretching, aerobic exercise, and resistance training was associated with preservation of exercise capacity and strength training volumes during the waitlist period. Participants spent 1.5 to 2 h training, 3 times a week, for a period of almost 4 months to attain that result [53]. Similarly, Florian et al. prospectively explored the effect of exercise training (warm-up, muscle strengthening exercises, and aerobic exercises) on exercise capacity and health-related quality of life in lung transplant candidates. In total, 58 participants underwent three 90-min sessions every week for 3 months. The 6-min walking distance significantly increased after completion of the program, compared to before, and components of quality of life showed improvements [54]. Wickerson et al. examined the effect of a supervised, thrice weekly prehabilitation program for the duration of the waitlist period. Prehabilitation consisted of 90-min sessions including stretching, functional exercises, resistance training, and aerobic exercise. They found that the 5-sit-to-stand test improved significantly in pre-frail/frail patients who completed 6 weeks of the prehabilitation program (pre: 11.4 ± 4.4 vs. post: 9.8 ± 2.8 s, *p* = 0.007) [55].

Hybrid prehabilitation designs are also tested in lung transplant candidates. Pehlivan and colleagues performed a prospective study consisting of at least 8 weeks of prehabilitation which included 2 days of supervised group exercises and 3 days of unsupervised at-home exercises per week. Significant improvements were observed after the program regarding 6-min walking test and dyspnea. Furthermore, health-related quality of life and symptoms of depression improved significantly [56]. This research group also published the results of their RCT examining inspiratory muscle training in the same year. Like the study described above, patients in the control group underwent a 5-day-per-week exercise program consisting of 2 days of supervised- and 3 days of unsupervised exercises for 3 months. The exercises focused on aerobic training, strengthening/resistance training, and breathing exercises. The intervention group underwent the same program as the control group with an additional inspiratory muscle training program. This training was performed 15 min, twice a day, 5 days a week for 3 months. The intervention group had significantly increased in 100-m walking distance, alveolar volume ratio of carbon monoxide diffusion capacity, and maximum inspiratory pressure. Furthermore, both groups demonstrated a decrease in dyspnea score [57].

Jastrzebski et al. investigated the effect of a combined inpatient home-based prehabilitation in a quasi-experimental study. Their study comprised of an intensive 4-week in-patient program where participants took part in 30-min training sessions daily, followed by an 8-week home-based prehabilitation program. The program consisted of respiratory muscle training and cycling to the limits of the patient’s tolerance. Six weeks following the program, improvements were seen in the quality of life and sensation of dyspnea in patients [58]. Gloeckl and colleagues performed a 3-week, inpatient, RCT in 60 patients. The intervention consisted of interval training on a bicycle (5–6 days/week). The control group performed continuous training on a bicycle for the same duration. Interval training was associated with lower intensity of dyspnea, but no significant differences were seen between the two groups regarding exercise capacity [59]. A large retrospective study by Kenn et al. found differing results regarding inpatient prehabilitation. The multimodal prehabilitation program consisted of physical training (5 to 6 days per week yielding 25 to 30 sessions), with exercises aimed at endurance training, individual strength training, specialized breathing techniques, and controlled coughing exercises. These exercise sessions were accompanied by educational sessions (2 days per week for 1 h) where participants learned about and how to implement coping strategies, address self-management, and incorporate nutritional interventions into daily life where necessary. Furthermore, attention was paid to post-operative care such as post-operative immunosuppression and the psychosocial aspects of living with a lung transplant. This program significantly improved exercise capacity and health-related quality of life [60].

Research on the effect of home-based prehabilitation programs is also performed in the lung transplant candidate population. Massierer and colleagues aimed to describe the changes in 6-min walk distance in lung transplant candidates who participated in a home-based exercise program. One hundred and fifty-nine participants took part in the program. The intervention consisted of warm-up exercises, aerobic training, and strength training. A moderate correlation was found between the 6-min walking distance prior to transplant and after transplant [61]. Overall, prehabilitation in lung transplant candidates seems to be effective.

### Prehabilitation and Liver Transplantation

The evidence for prehabilitation in liver transplant candidates is extensive. A recent systematic review highlighted the positive effects of prehabilitation regarding safety and feasibility in this population [62••]. Eight studies were included in the systematic review including three RCTs and five cohort studies. Limongi et al. performed a small (intervention arm *n* = 5) RCT with the aim to evaluate the effects of an inspiratory muscle training program in liver transplant candidates. The intervention group participated in home-based diaphragmatic isometric exercise, diaphragmatic breathing exercises, lifting upper limbs with a bat, and strengthening the abdominals. Significant improvements were found in forced expiratory flow in the intervention group [63]. Another small (intervention arm *n* = 11) RCT aimed to investigate the safety and feasibility of a 2-month exercise program. The intervention consisted of aerobic training and resistance-strength exercise, supervised and unsupervised, thrice a week. Significant improvements in 6-min walking distance were seen after the program but no changes were found regarding hand grip strength [64]. One small (intervention arm *n* = 9) RCT assessed the benefits of a 12-week, multi-modal, home-based physical activity program in liver transplant candidates. The intervention consisted of biweekly counseling sessions to increase activity and participants were given 12 g of an essential amino acid supplement daily. Significant improvements in functional exercise capacity were seen in the intervention group according to the 6-min walk test but not in peak aerobic capacity from cardiopulmonary exercise training [65].

Cohort studies show similar results in the liver transplant population. One prospective study, which included eight participants, evaluated the acceptability of a 12-week, supervised, personalized, adapted physical activity program and its impact on quality of life, aerobic capacity, and muscle strength. Significant improvements were seen after 12 weeks regarding aerobic capacity and muscle strength, quality of life remained unchanged [66]. Another prospective, 12-week study which involved a home-based, unsupervised exercise program, consisting of functional resistance exercises, aerobic exercises and a walking program (10 min of walking), 3 times per day, daily, and 20 min of exercise twice a week. Aerobic capacity improved significantly after 6 weeks and after 12 weeks. Improvements in functional capacity as measured by the short physical performance battery were only seen after 12 weeks [67]. Another prospective study, examined the effect of a supervised physical training on 16 liver transplant candidates. Participants in the intervention group underwent aerobic training thrice weekly for a duration of 6 weeks. Nine out of 16 participants completed the entire program and peak oxygen consumption significantly increased in the intervention group [68]. Al-Judabi et al. performed a large (intervention arm = 258) retrospective study on the effect of an in- and outpatient exercise training program for liver transplant candidates. The intervention included nutritional support and both supervised training at the hospital gym and unsupervised training at home. The program consisted of 1 to 5 sessions weekly consisting of aerobic training, resistance strength exercise, and education lasting until suitable for transplantation. The control group received care as usual. No significant differences in post-operative complications were found between the two groups. However, a trend in lower readmission rate and shorter length of stay was seen in the intervention group [69]. Lin et al. performed an ambispective cohort study on 517 liver transplant candidates. Participants received an exercise prescription and one dietary consultation and were asked to train for 30 min, five times per week, until transplantation. The training was primarily done at home, unsupervised, with a follow-up appointment once a month. Significant improvements of the liver frailty index were found after training, and a larger effect was seen in patients who adhered to more than 80% of the exercise sessions until transplantation [70]. Overall, 6 out of 8 studies demonstrated significant improvements in aerobic or physical capacity in liver transplant candidates.

### Prehabilitation and Heart Transplantation

A small number of studies explored the effect of prehabilitation in heart transplant candidates. In 2017, Gimeno-Santos et al. performed an exploratory pilot study spanning 2 years which examined the effect of multi-model prehabilitation on clinical outcomes in heart transplant candidates. Eleven patients completed the 8-week prehabilitation program which consisted of 1-h sessions, 2 sessions per week at the outpatient gym facility located at the transplant center. The program was multimodal in nature with one part of the program focused on improving physical functioning through endurance- and strength training, nutritional supplementation when necessary, and weekly mindfulness sessions aimed at stress reduction. After 8 weeks, significant improvements were in seen in functional status, exercise capacities, physical activity, quality of life, and level of anxiety. Furthermore, no adverse events occurred, and overall adherence was 86%. Nine out of 11 patients underwent heart transplantation after completing the program, and these patient has an uneventful post-operative period [71]. Unlike the study above, Taya et al. examined the effectiveness and safety of supervised, in-patient prehabilitation in heart transplant candidates. The intervention initially consisted of aerobic exercise training. Once participants were able to perform aerobic exercise for 15 min without a significant increase in rate of perceived exertion, high-intensity interval training (HIIT) was initiated. The HIIT intervals were performed for 1 min, with 4 min of rest in between, repeated 3–4 times. The intervention took place 3–4 times per week. The duration of aerobic training and HIIT were 47.0 ± 23.6 and 27.8 ± 13.2 days, respectively. Furthermore, knee extensor strength significantly increased during HIIT (4.42 ± 1.43 vs. 5.28 ± 1.45 N/ kg, *p* < 0.001). No changes in handgrip strength were measured throughout the program [72]. Similarly, Forestieri and colleagues, sought to evaluate the effect of an inpatient, cycle ergometer exercise program on exercise capacity and inspiratory muscle function in patients with heart failure awaiting heart transplantation. The control group (*n* = 11) underwent sessions focused on breathing exercises and global active exercises whereas the intervention group (*n* = 7) exercised on a stationary cycle ergometer in an upright position for 20 min, alternating between 3 min of cycling followed by 1 min of rest. Both groups demonstrated an increase in 6-min walk test after the intervention compared to baseline; however, only the intervention group showed a significant increase. Regarding the inspiratory muscle strength evaluation, significant improvements were seen in the intervention group as well. [75] The lack of evidence regarding prehabilitation in patients awaiting heart transplantation has been attributed to the high degree of frailty in this population [71, 74]. Furthermore, patients may be unable to train due to cardiac failure itself. However, the above-mentioned studies show promising results regarding safety and feasibility for this population.

## Limitations of Current Prehabilitation Research and Future Directions

There is a great degree of heterogeneity of the prehabilitation studies performed in SOT candidates. First, the length of prehabilitation programs ranges from 5 to 16 weeks, or for the entire length of the pre-transplant waiting period, which makes it difficult to compare studies and determine overall efficacy. Second, prehabilitation program modalities differ across but also within SOT groups. While telerehabilitation programs for prehabilitation grew during the COVID-19 pandemic, many hospitals still opt for an in-person approach. Regarding multimodal and unimodal approaches, both methods are shown to be effective. However, multimodal prehabilitation, comprising physical training, nutritional support, and psychologic support, addresses the interplay between physical and psychological factors and is known to positively influence the outcomes of an intervention [75, 76••]. Therefore, we recommend a multimodal approach for prehabilitation in SOT candidates. Overall, consensus needs to be reached regarding the optimal prehabilitation regimen for SOT candidates.

Other limitations of current research include small sample sizes and the lack of sufficiently powered RCTs. This may be due to the unpredictable nature of the pre-transplant period. However, large, high-quality trials are needed to reduce selection bias in the population and to determine the cause-effect relationship between prehabilitation and functional outcomes. With respect to the population, often the most fit patients are selected for the intervention, while frail individuals will probably benefit the most. If patients do not improve due to/with prehabilitation this could be an indication that their resilience is limited. In that case, a comprehensive geriatric assessment to evaluate those patients or reconsideration of the indication for transplantation might be wise. Regarding outcomes, most studies examined the effect of prehabilitation on functional outcomes such as aerobic capacity and muscle strength. Though these outcomes are important for preoperative functional status and post-operative recovery, the effect of prehabilitation on pre- and post-transplant clinically relevant outcomes such as ICU and hospital length of stay, graft function, complications, mortality, and readmissions should be determined. When this is known, transplant centers will be more inclined to implement prehabilitation programs into clinical practice, if shown to be effective. Also, to combat barriers to implementation, future studies should focus on self-management, exercise education, and other aspects related to behavioral change as prehabilitation demands commitment from patients. Solid organ transplantation is a life-changing event which may motivate patients to improve their lifestyle; however, it is important that this change is maintained after transplantation. Furthermore, the economic burden of the implementation of a prehabilitation program should be studied. Together, this evidence will uncover how to best deliver a prehabilitation program for SOT candidates.

## Conclusions

In conclusion, prehabilitation is an effective method for improving aerobic fitness in kidney-, lung-, liver-, and heart transplant candidates which is especially important in the context of the aging population. Future research is needed to uncover the effects of prehabilitation on pre- and post-transplant clinical outcomes.
